# Community-Driven Health Education: Role of Health Literacy Seminars in Empowering Underserved Populations and Medical Students

**DOI:** 10.7759/cureus.106644

**Published:** 2026-04-08

**Authors:** Ali A Raza, Pruthvi Patel, Sireen S Hilo, Mary O Smith, Greg Jacobs

**Affiliations:** 1 General Surgery, Henry Ford Health System, Detroit, USA; 2 Diagnostic Radiology, University of Mississippi Medical Center, Jackson, USA; 3 Pediatrics, Southeast Health Medical Center, Dothan, USA; 4 Internal Medicine, Alabama College of Osteopathic Medicine, Dothan, USA; 5 Emergency Medicine, Alabama College of Osteopathic Medicine, Dothan, USA

**Keywords:** addiction, health seminars, medical education, preventive medicine, public health, rehabilitation

## Abstract

Health literacy has increasingly been recognized as a crucial determinant of health outcomes, specifically among underserved populations facing immense barriers to accessing and understanding their healthcare plans. This research paper advocates for community-based health literacy seminars to address this disparity. These seminars can help underserved populations connect with individuals involved in healthcare within their communities while also providing experiential learning opportunities for medical students, fostering the growth of communication skills and cultural competence. The study aimed to evaluate the efficacy of a health seminar series provided to a rural underserved community, while also examining the reciprocal benefits experienced by medical students involved in this endeavor.

Through collaboration with community partners, a series of health literacy seminars was conducted, featuring interactive sessions encompassing diverse health topics specifically tailored to the needs of this population. These sessions were designed to facilitate engagement and relationship-building exercises. The seminars also provided medical students with the opportunity to participate in their community while offering support, sharing knowledge, and building positive physician-patient relationships.

Pre- and post-seminar surveys were used to gauge changes in participants' perceived health literacy levels. Significant improvements in literacy scores were observed throughout this seminar series across a variety of topics. This quantitative data was complemented by qualitative discussions with participants and students to understand why these topics were chosen, how the seminar impacted those involved, and why it is important to continue making personal health knowledge more accessible throughout the community. Both participants and students expressed feelings of empowerment and confidence, underscoring the practical benefits of the seminar.

Active engagement with a diverse community can provide students with firsthand insight into the unique challenges faced by the individuals they serve while also benefiting the members of the community. Results obtained from the pre-series and post-series surveys notably demonstrated a 47.47% increase in male participants' perceived ability to implement strategies that would allow them to take better care of their overall health and well-being. Results also indicated a 47.20% increase in female participants' perceived awareness of resources available to help manage their stress.

In conclusion, this study underscores the benefits that health literacy seminars bring to the medical education of students while also improving the health literacy of the communities they serve. By fostering reciprocal and experiential learning, these seminars serve as a key initiative for addressing health disparities and promoting health equity. This paper encourages future efforts to prioritize community-based interventions and integrate real-world learning opportunities into medical school curricula to cultivate an empathetic and culturally competent healthcare network.

## Introduction

Health literacy refers to the set of skills that “determine the motivation and ability of individuals to gain access to, understand, and use information in ways that promote and maintain good health” [1]. In other words, health literacy is necessary for making informed decisions about one’s health and is intricately linked to health outcomes. Individuals with lower levels of health literacy experience increased hospitalizations, costs, and worse overall health status [2]. Therefore, improving health literacy is crucial for achieving health equity and reducing disparities for all.

Health literacy is an essential component of healthcare access and outcomes. However, in past years, it has also become a barrier for those unable to fully understand their health conditions and healthcare plans. Around the world, individuals, especially in underserved areas, struggle with understanding their own health and how they can take the next best steps to improve it [3]. While in medical school, students are taught various pathologies and pharmacologic treatments, little emphasis is given on how to articulate medical language in a way that patients of all health literacy levels can understand.

To address these challenges, we conducted a health literacy seminar series in a specific underserved community, providing accessible education to individuals with limited access to healthcare services. This initiative was paired with a research project that evaluated its impact, demonstrating an increase in participants’ subjective understanding of their health. This was assessed through several pre- and post-seminar surveys that quantified each participant's confidence regarding the aspects discussed during the seminar. Additionally, the program highlighted the value of experiential learning, as medical students involved in the seminars strengthened their communication skills and ability to translate complex medical concepts into patient-centered language. This paper aims to explore the complex nature of health literacy, how it impacts individuals and communities, potential interventions and their benefits, as well as how this can improve medical students' experience via experiential learning.

Underserved communities, which may include a disproportionate number of racial and ethnic minorities and individuals from low socioeconomic status (SES) backgrounds, face increased barriers to healthcare services as well as challenges in understanding their personal health information [4]. These barriers are rooted in systemic inequities, which contribute to a cycle of low health status and illness [3]. Improving these conditions requires healthcare workers and leaders to recognize and address these disparities to ensure equitable access to healthcare for all individuals, regardless of SES or background [4].

Community-based interventions, such as health literacy seminars, offer a way for medical students or other healthcare workers to connect with their community [5]. They can build a positive physician-patient relationship while also improving their didactic knowledge by applying it to real-world scenarios. Engaging small local communities with health seminars tailored to their specific needs encourages patients to take an active role in their healthcare [6]. Participants can equip themselves with the necessary knowledge to advocate for their health and the tools needed to access care. By creating and disseminating accessible and relevant health education, students can take control of what they learn in the classroom and see how it applies to a diverse population [6].

Medical students are expected to draw their knowledge from a wide variety of resources, but they do not always have access to learning opportunities in real-world communities until their clinical years. Health seminars are just one approach through which students can provide value to their community. Experiential learning and seeking knowledge from the communities they wish to serve are immensely valuable experiences for becoming well-rounded physicians [7]. These experiences enhance students' understanding of the diverse needs and experiences of patients while reinforcing their commitment and passion to serve as advocates for health equity. Medical students have the ability to bridge the gap between providers and patients, influencing a more positive impression of the healthcare system and encouraging patients to have open and honest conversations with their providers [5]. These experiential learning instances deepen students' appreciation for the societal factors that ultimately influence health outcomes and further inspire careers dedicated to addressing these disparities [8].

The role of the physician in a patient’s health literacy is not well-defined. Some physicians consider it their responsibility to ensure that patients understand the implications of their health and treatment. Other physicians view a patient’s low health literacy status as a result of that patient’s lack of effort or as their own fault [9]. With this program, we aimed to create further motivation among medical students to do everything possible to improve patient health literacy, in addition to learning ways to teach and explain concepts to patients with low health literacy in practice.

The students who participated in this program reported a deepening of their understanding of the challenges that face their local community and underserved populations. The exploration of healthcare through the patient’s lens provided a unique perspective that is crucial early on in one’s career, allowing students to gain empathy that is often lost in healthcare workers as they move through their grueling careers. Students were able to witness firsthand the change that these seminars brought to their local communities. These seminars can be a catalyst for their passion to change the healthcare system and provide access for a wider array of individuals, as well as their passion for bringing shared informed decision-making into their practice.

In summary, both students and the community can benefit from students engaging with their community and providing health literacy knowledge to an underserved population. For students, it serves as an opportunity to strengthen what they learn in the classroom while also reigniting their passion for being of service to others. Engaging with the population early on in their careers fosters a deeper appreciation for the diverse array of patients they will encounter. Additionally, it serves the community by bridging a gap that previously existed. Speaking with students who are training to become physicians helps the community connect with their local healthcare resources and become more accepting and trusting of the healthcare institution [4]. By providing the community with the tools necessary to advocate for themselves and the knowledge to understand their conditions and treatment plans, we can lead to improved health outcomes and enable these individuals to live longer, more fulfilling lives.

## Materials and methods

Ethical approval

Ethical approval to conduct the study was obtained from the Alabama College of Osteopathic Medicine Institutional Review Board (IRB). IRB approval was obtained prior to the beginning of the seminar series (IRB#HS230130-E-1).

Recruitment

To be eligible for inclusion in the study, participants were required to be current residents of the ARK of Dothan, Alabama, USA, and actively enrolled in the ARK Transitional Program at the time of data collection. This population consisted of, but was not limited to, individuals who had previously been incarcerated, lacked stable access to housing, or were undergoing drug rehabilitation. Participants needed to be 18 years of age or older and capable of providing informed consent. Fluency in English was also required to ensure comprehension of seminar content and facilitate participation in follow-up discussions or data collection tools, such as surveys or interviews. The sample population consisted of 29 individuals - 14 self-identified male and 15 self-identified female participants. These participants were able to attend the seminars in their entirety during the data collection process. Individuals were informed one week in advance that a health seminar would be held, as well as the topic that would be discussed. The number of individuals in each seminar varied depending on individual schedules or if there were new residents. Participation was optional, and there was no monetary incentive for attending the health seminars. Individuals were excluded from participation if they were not enrolled in the ARK Transitional Program or not residing at the ARK of Dothan during the study period. Residents who were under 18 years of age, unable to provide informed consent, or who chose not to participate voluntarily were also excluded. Additionally, transient individuals who were not present for the full duration of a seminar session were not included in data analysis to maintain the consistency and integrity of the data.

Duration

This project was conducted from March 2023 to July 2024. Data analysis for the project was carried out between July and September 2024.

Data collection

This study was initiated by distributing a preliminary survey assessing the overall health literacy of the ARK residents. The survey was composed of 10 health literacy statements, which the residents rated from 1 to 5 on a Likert scale (1 indicating complete disagreement and 5 indicating complete agreement) [10]. This preliminary survey can be found in Appendix A. Along with the survey, an informed consent statement was provided, reminding individuals that this data collection was optional and would not affect their participation in the seminar. During this initial meeting, a verbal survey was conducted regarding which health topic the residents wanted to learn more about, leading to the creation of a basic topic list for the following few months. Those who attended a minimum of eight seminars were eligible to complete a post-series survey, identical to the preliminary survey, to assess the overall impact of the project. 

Each seminar lasted approximately 45 minutes and was hosted by two medical students involved in the planning of the event, along with one medical student who demonstrated proficient knowledge regarding the topic. All medical students present participated in the creation of the seminar materials and the discussion. For example, to be eligible to host the Heart Health seminar, all medical students involved must have passed the cardiovascular systems course, and one student must have served as the committee chair for the cardiovascular interest group of the school. The outline for the seminar was subsequently reviewed by a licensed physician on the faculty of the medical school. During each health seminar, a pre-survey and a post-survey were provided. These were identical documents consisting of six questions aimed at gauging each resident's confidence in their health and were used to assess the efficacy of each seminar on the health literacy of the residents. Each survey was consistent across the project and focused on the specific health topic of that seminar. An example of these surveys can be found in Appendix B.

At the end of each seminar, there was also an open discussion session allowing the residents to ask questions and speak freely in a safe and nonjudgmental environment. All surveys were collected, and data were manually input into Microsoft Excel (Microsoft Corp., Redmond, USA) for further analysis.

Some seminars included an interactive component or activity. For example, during the Women’s Health seminar, breast models with sample pathologies of breast cancers and tumors were used to teach residents how to perform important at-home self-examinations. During the seminar on Narcan use, a sample of Narcan was shown, and its use was demonstrated by a volunteer licensed pharmacist. In the Nutrition seminar, foods from all food groups were brought in to share a nutritious and balanced meal among the participants while learning.

In addition to considering the responses from the preliminary survey, seminar topics were chosen based on significant public health issues. The various seminars delivered were themed around cardiovascular health, mental health, women’s health, sun and heat safety, sleep and caffeine hygiene, nutrition, physical activity, insurance, patient advocacy and personal healthcare experiences, and Narcan and addiction.

Data analysis

**Figure 1:**
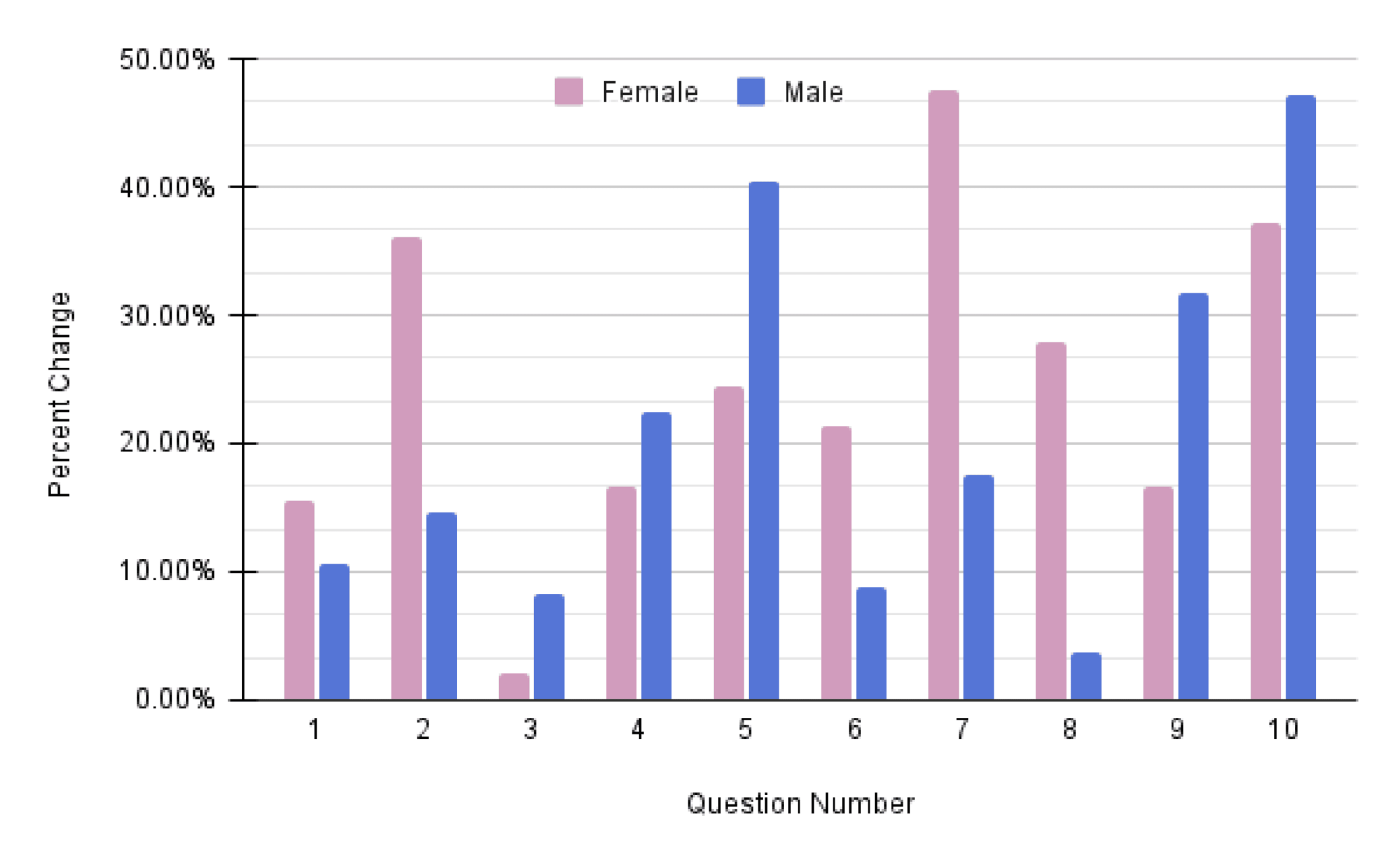
Overall healthcare seminar series data Question number refers to the question set presented in Appendix A.

Data were transcribed into Microsoft Excel, where the averages of the pre-survey and post-survey were calculated for each seminar. Results were viewed as an aggregate for both male and female groups, as well as individually within same-sex groups. A percent change was calculated for the pre- and post-survey results by finding the absolute value between the two data points and then using the pre-survey data point as the denominator. Bar charts of this percent change were created to determine which aspects of the health seminar were most effective and which were the least effective. The analysis of the overall healthcare seminar series effects is presented in Figure 1.

Limitations 

One limitation of this study is the relatively small and specific sample size. These participants were all residents of the ARK Transitional Program, which is limited to those who were previously incarcerated, lacked stable housing, or were in drug/alcohol rehabilitation. While this unique population may offer a unique and valuable perspective, these findings may not be generalizable to other groups with differing health literacy backgrounds or socioeconomic factors. Furthermore, the variation in resident schedules somewhat limits the comparison between seminars, since there may be a variation in the concentration of individuals who belong to specific socioeconomic or demographic groups.

Another limitation includes the use of self-reported surveys to assess health literacy. While these surveys provided insight into the confidence participants had in managing their personal health, this is a subjective response that may not fully assess the objective improvement in health knowledge. Response bias is a potential issue, as participants may have over- or underestimated their understanding. Finally, the absence of long-term follow-up on specific seminars means that this study cannot assess the change in behaviors that participants undergo due to attending these seminars.

## Results

Over 13 months, 11 health seminars were hosted to improve health literacy among participants. Each seminar featured similar health questionnaires designed to assess changes in health literacy over the course of the program. An average of 25 participants attended each health seminar during this period. Among the attendees, an average of 52% were female (n=13) and 48% were male (n=12) individuals. The age distribution ranged from 21 to 62 years, with a median age of 36. The age distribution was as follows: six individuals (24%) aged 21-29, 15 individuals (60%) aged 30-49, and four individuals (16%) aged 50-62.

The questionnaires assessed health literacy through a series of questions that measured understanding of various health concepts, relationships with medical professionals, and personal confidence in using healthcare systems and managing personal health issues. The data showed an improvement in individual confidence in health literacy across all seminars, irrespective of age or gender.

Of those who attended eight or more seminars, the average pre-series score for participants' perceived ability to "implement strategies now to take better care of (their) overall health and well-being" was 50.7% (2.54 out of 5), while the average post-series score for the same question was 72.2% (3.61 out of 5). This represents a 42.1% average increase in a participant's perceived ability to implement strategies to improve their health.

**Figure 2:**
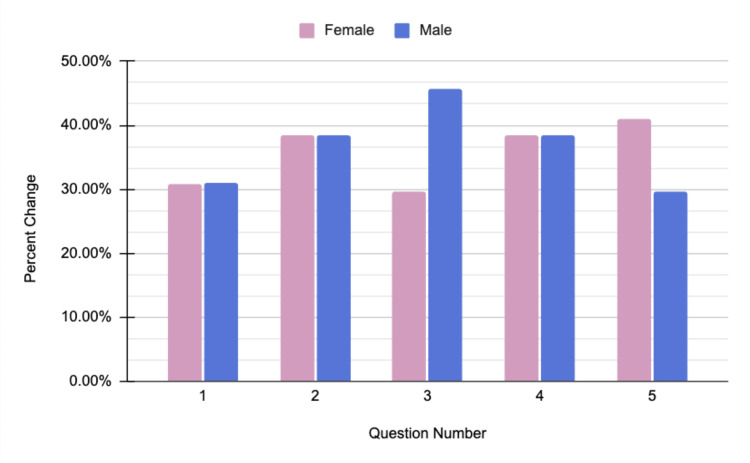
Narcan health seminar data Questions asked on the survey: 1. I am confident in my knowledge about Narcan and how to use it. 2. I know the signs and symptoms of when to administer Narcan. 3. I know how to store Narcan and where to obtain it. 4. I know how to recognize a positive response to Narcan administration. 5. I know the steps to take after administering Narcan.

While participants exhibited an increase in confidence in their knowledge and ability to seek help for all health seminars, there was a difference between genders regarding which topic they gained the most confidence in. Male individuals showed the largest single-question difference before and after the health seminar regarding Narcan use. When asked if "(they) know how to store Narcan and where to obtain it," they had a pre-seminar score of 53.8% (2.69 out of 5) and a post-seminar score of 78.4% (3.92 out of 5), representing an average increase of 45.7% in confidence about how to obtain and store Narcan. Figure 2 highlights the results of the Narcan seminar.

**Figure 3:**
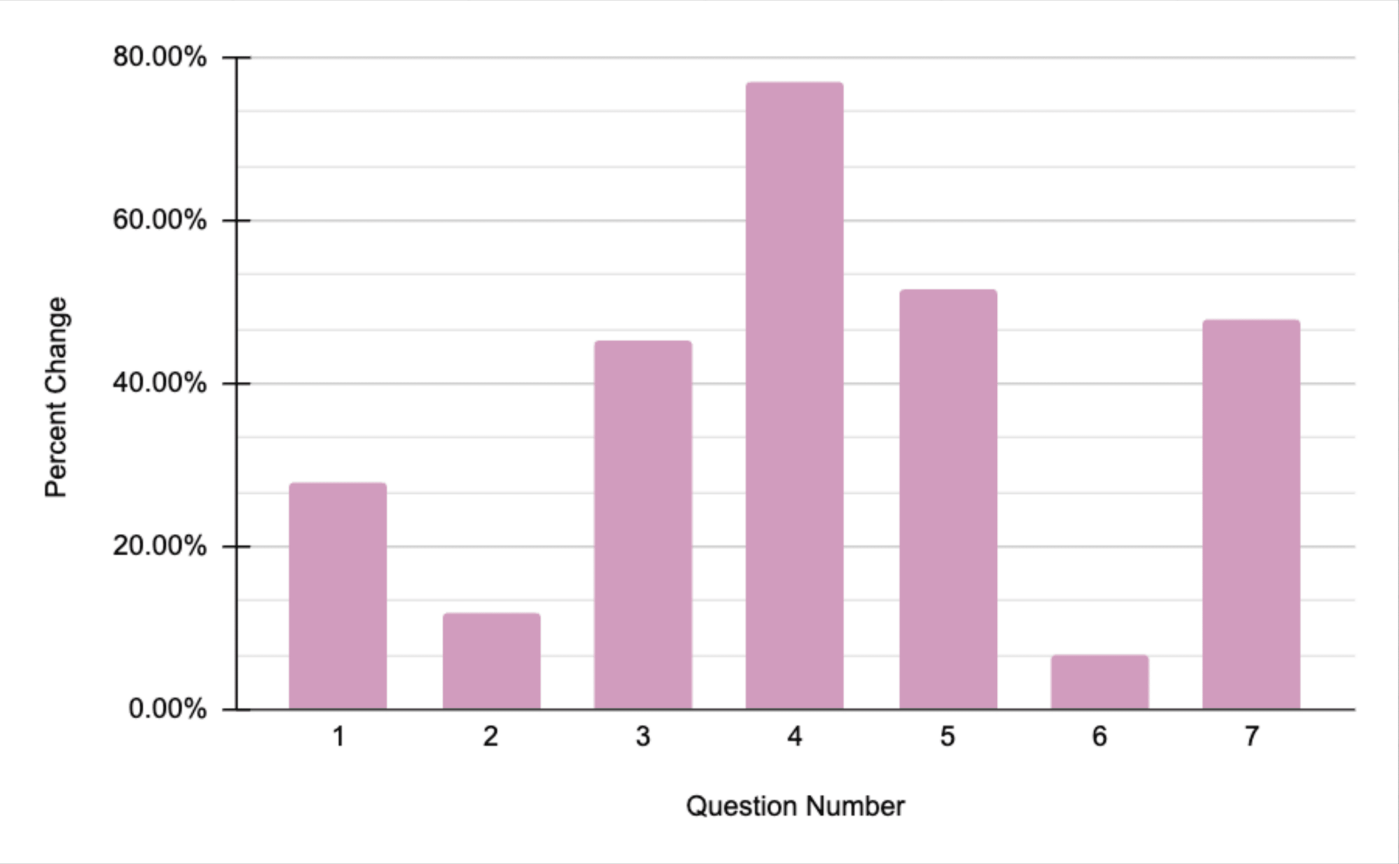
Women's health seminar data Questions asked on the survey: 1. I am confident in my knowledge about women's health. 2. I am aware of practices to prevent sexually transmitted infections (STIs). 3. I know the symptoms of menopause. 4. I am familiar with strategies for managing menopause. 5. I know the different screenings available for women throughout their lives. 6. I am aware of the various birth control options available. 7. I know practices to help prevent osteoporosis.

Female individuals had the largest difference before and after the health seminar regarding Women's Health. When asked if "(they) know practices to help prevent osteoporosis," the average pre-seminar score was 58% (2.90 out of 5) and the post-seminar score was 86% (4.30 out of 5). This represents an average 48.3% increase in a female participant's confidence in their knowledge of preventing osteoporosis. Figure 3 highlights the data gathered from the Women's Health seminar.

**Figure 4:**
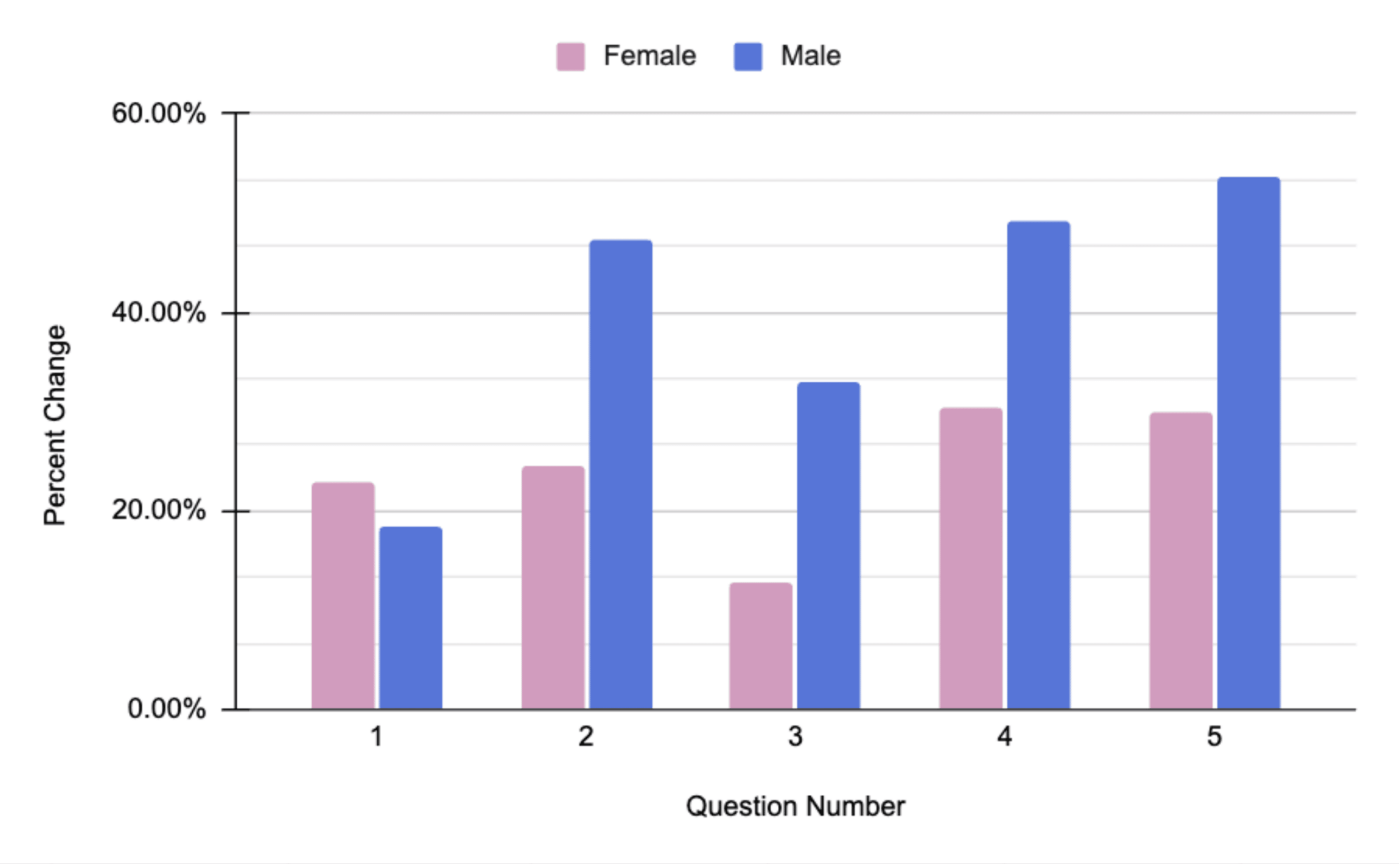
Sun and heat safety seminar data Questions asked on the survey: 1. I am confident in my knowledge of sun and heat safety. 2. I can recognize the signs of skin damage and skin cancer. 3. I know the signs of heat illness, such as heat exhaustion and heat stroke. 4. I know the appropriate actions to take in a heat illness emergency. 5. I understand what it means to be water competent and how to respond in a water-related emergency.

Participants also showed improvements in their ability to identify potential warning signs and symptoms of various conditions, including myocardial infarctions, heat stroke, opiate overdose, and depression. In response to the statement “I know the signs of heat illness, such as heat exhaustion and heat stroke,” male participants had an average pre-seminar response of 66.2% (3.31 out of 5) and an average post-seminar response of 88% (4.40 out of 5). For female participants, the pre-seminar response was 78.8% (3.94 out of 5), and the post-seminar response was 88.8% (4.44 out of 5). Figure 4 highlights the data gathered during the Sun and Heat Safety Health seminar.

There was some variation in the questions asked, as well as in the data gathered from the male and female participant groups, across the remainder of the health seminars. While these results are promising, they should be interpreted with caution, given the limitations related to sample size and study design. Additionally, self-reported data may be subject to bias, which could influence participant responses.

## Discussion

The findings uncovered throughout this pilot program highlight the impact and implications that improving a community's health literacy has on both underserved populations and medical students. Throughout this experience, medical students were able to engage with their community early in their medical education and apply their didactic knowledge to clinically relevant information that benefitted their community while simultaneously promoting health equity and addressing healthcare disparities. As seen in the results, participants showed significant improvements in their confidence levels and knowledge across various health topics following each seminar. Furthermore, exposure to healthcare workers in a low-stress environment promoted a positive relationship between underserved communities and healthcare institutions. These positive relationships encouraged these individuals to have open and honest conversations with their physicians and other healthcare providers. These improvements suggest that targeted community-based interventions have the potential to empower underserved populations with the skills and information necessary to make informed decisions regarding their health.

The use of pre- and post-surveys allowed for the assessment of the impact that each health seminar had on participants' confidence in their health literacy levels [11]. Each seminar demonstrated an improvement in confidence levels through the dissemination of health knowledge, highlighting the effectiveness of the intervention while addressing the specific needs of the underserved populations. By personalizing the content of each seminar to align with the needs and preferences of participants, this pilot program shows how a patient-centered approach can serve to improve patient-physician relationships while also improving health outcomes.

The decline of empathy in medical students throughout their medical journey is a concern that has been investigated in recent years [6]. As students advance through the rigorous and stressful pre-clinical and clinical years, it has been found that they are prone to being “desensitized” to the clinical and social conditions of patients [12]. This desensitization only worsens as students graduate and enter long careers as practicing physicians. Many efforts have been discussed among schools to prevent this loss of empathy [7]. This community-focused pilot program emphasizes early collaboration between medical students and the community in which they reside. This early exposure can potentially address the loss of empathy in medical students that is unfortunately increasing.

Longitudinal studies that track sustained improvements in health literacy over a several-year period would further benefit the results of this pilot program. Follow-up surveys occurring over months to years, instead of immediately after, would help to provide valuable information regarding long-term knowledge retention. Objective measurements would also help further expand upon habit transformation and lifestyle modification. Similar programs and studies could further assess the effects that improved health literacy, lifestyle, and habits have on these populations and show how their overall well-being changes over time.

Moreover, medical student engagement with underserved populations may help garner interest in pursuing careers working with rural and underserved groups. There is a shortage of United States (US) physicians working in primary care and in rural areas, and volunteerism during medical school in such settings has been shown to factor into the decision to pursue these types of careers [8].

Finally, this study’s results highlight how important health literacy interventions are in promoting health equity in underserved communities. By establishing personal relationships with these populations and healthcare workers early in their careers, both can benefit. Underserved populations can improve their health literacy in a safe environment while also developing more open and comfortable relationships with their physicians. Medical students are better able to understand how their didactic knowledge applies to clinical information while also learning more about their personal biases and how they can better manage diverse patient care. Moving forward, efforts that continue to address healthcare disparities in underserved communities through collaboration and community-driven approaches are necessary for furthering health equity and improving the overall well-being of diverse populations.

However, it is important to acknowledge the limitations present in the study design, sample characteristics, and reproducibility of results. The relatively small sample size and common characteristics of the participants limit the generalizability of the results to a larger community, and the specific challenges faced by this group must be extrapolated to fit the general population of the US. Furthermore, the reliance on self-reported data presents an opportunity for personal biases to enter and influence the accuracy of results. Future studies would benefit from employing larger and more diverse samples, as well as more objective measurements to corroborate self-reported outcomes. Additionally, conducting surveys over months to years will help assess long-term information retention and provide valuable data points to determine whether health seminars are truly effective.

## Conclusions

In society, physicians are often viewed as role models, especially in the area of health maintenance. Having future and current physicians lead community health seminars to promote health literacy can improve the overall health of a community. Targeting these efforts to at-risk populations, such as those at the ARK - whose program residents are transitioning from homelessness, prison, or drug rehabilitation - can bring much-needed health services to the groups that need them the most. Oftentimes, these groups are neglected in healthcare and health literacy efforts, resulting in new or worsening chronic conditions. Programs such as this, which focus on addressing health literacy gaps, aim to instill a passion for improving community health in medical students early in their education. We hope they will continue to promote health literacy as medical professionals as well. These long-lasting efforts will increase attention toward preventative health, hopefully decreasing mortality and long-term complications in these groups. 

Our group continues to deliver these health literacy seminars at the ARK. Because most residents stay in the program for an average of 12 months, seminar topics are delivered annually to reach those who are new to the program and have not previously participated in our seminars. This initiative will continue with classes of medical students below us, with each seminar building on the topics covered in the previous year. Other subjects we aim to explore in the seminars include dental health, seasonal illnesses, men’s health, and personal hygiene. Additionally, we aim to make the seminars more hands-on and incorporate more activities that facilitate learning, as we have attempted to do with a few of the seminars. If there are any residents who attended the seminar the previous year, we hope to assess their prior knowledge and retention of the topic and build on what they have learned.

## Appendices

Appendix A

**Table 1:** Initial preliminary health seminar survey questions Table created by the authors.

No.	Question
1	I understand the importance of a balanced approach to maintaining good health.
2	I know how to access resources to help maintain good health.
3	I recognize the benefits of regular physical activity for overall well-being.
4	I am aware of the impact that proper nutrition has on health and energy levels.
5	I know how to access resources that will allow me to take in proper nutrition.
6	I know how managing stress contributes to better health outcomes.
7	I am aware of resources that will better help me manage my stress.
8	I recognize the importance of regular health check-ups and preventive care.
9	I understand the connection between mental and physical health and its impact on overall well-being.
10	I am able to implement strategies now to take better care of my overall health and well-being.

Appendix B

**Table 2:** Heart health seminar pre- and post-survey questions Table created by the authors.

No.	Question
1	I am confident in my understanding of heart health.
2	I prioritize my heart health in my life.
3	I am aware of and am able to seek out resources available for heart health support.
4	It is important to seek out help for heart health concerns.
5	Those with heart health concerns are able to recover and live a fulfilling life.
6	I am aware of how stress can affect my heart health.
